# Utilization of engineered resistance to viruses in crops of the developing world, with emphasis on sub-Saharan Africa

**DOI:** 10.1016/j.coviro.2017.07.022

**Published:** 2017-10

**Authors:** Jan F Kreuze, Jari PT Valkonen

**Affiliations:** 1 International Potato Center, Lima 12, Peru; 2 Department of Agricultural Sciences, University of Helsinki, FI-00014 Helsinki, Finland

## Abstract

Viral diseases in crop plants constitute a major obstacle to food security in the developing world. Subsistence crops, including cassava, sweetpotato, potato, banana, papaya, common bean, rice and maize are often infected with RNA and/or DNA viruses that cannot be controlled with pesticides. Hence, healthy planting materials and virus-resistant cultivars are essential for high yields of good quality. However, resistance genes are not available for all viral diseases of crop plants. Therefore, virus resistance engineered in plants using modern biotechnology methods is an important addition to the crop production toolbox.

## Highlights

• Genetic engineering of virus resistance is feasible in monocots and dicots.
• Engineered virus resistance can be successful in all major crops.
• Developing countries are building capacity in plant biotechnology for crop improvement.
• Integration of viral sequences in plant genomes is a natural process.
• Definitions and regulations of transgenic plants require reassessment.

**Current Opinion in Virology** 2017, **26**:90–97

This review comes from a themed issue on **Engineering for viral resistance**

Edited by **John Carr** and **Peter Palukaitis**

For a complete overview see the Issue and the Editorial

Available online 8th August 2017

**http://dx.doi.org/10.1016/j.coviro.2017.07.022**

1879-6257/© 2017 The Authors. Published by Elsevier B.V. This is an open access article under the CC BY license (http://creativecommons.org/licenses/by/4.0/).

## Introduction

Viral diseases constitute a major threat to crop production worldwide. The problem is exacerbated in developing countries, particularly in tropical areas where crop-free seasons are rare and plants are under continuous pressure from viruses transmitted by vectors from both cultivated and wild plants [1]. Africa suffers from major recurring plant virus pandemics and epidemics in important crops. Well-known viral diseases caused by DNA viruses include cassava mosaic disease (CMD) caused by begomoviruses (members of the family *Geminiviridae*) [2^•^], banana bunchy top disease caused by a babuvirus (a member of the family *Nanoviridae*) [3] and maize streak disease caused by maize streak virus (MSV), a mastrevirus (a member of the family *Geminiviridae*) [4]. Cassava brown streak disease is caused by two closely related RNA viruses (members of the genus *Ipomovirus*, family *Potyviridae*) [5, 6]. In eastern Africa, maize suffers from lethal necrosis caused by co-infection by two RNA viruses, maize chlorotic mottle virus (a member of the genus *Machlomovirus*, family *Tombusviridae*) and the potyvirus sugarcane mosaic virus (a member of the family *Potyviridae*) [7]. Sweetpotato virus disease (SPVD), the most devastating disease of sweetpotatoes, is also caused by co-infection by two RNA viruses, the crinivirus *sweet potato chlorotic stunt virus* (SPCSV, a member of the family *Closteroviridae*) and the potyvirus sweet potato feathery mottle virus [8].

Natural sources of resistance to many viral diseases of tropical crops are known and deployed in breeding. In many cases, however, resistance sources are lacking, or the genetic complexity and difficulties in introgressing resistance genes to cultivars by crossing hamper the efforts of crop improvement. Therefore, development and transfer of resistance to crops by biotechnological means offers an attractive alternative solution.

The studies of Powell Abel *et al.* [9^••^] in the 1980s showed that transformation of tobacco plants (*Nicotiana tabacum* L.) to express the coat protein of tobacco mosaic virus made the plants resistant to this virus. Similarly, transformation of plants to express non-structural viral proteins [10] and truncated, defective viral genes also provided protection against homologous viruses [11]. These findings created much excitement and hope for a quick solution to viral disease problems in crop plants. The mechanism of resistance in genetically engineered plants became comprehensible with the discovery of post-transcriptional gene silencing (i.e., RNA silencing or RNAi) [12], which informed further improvements in construct design, such as binary vectors expressing virus-specific inverted-repeat (hairpin) RNA to target the virus to degradation by RNAi [13^•^].

It was later realized that some viral proteins suppress or interfere with antiviral RNAi [14••, 15] and virus-derived resistance could fail if the plant was infected with a virus that differed from the donor of the transgene by >15–20% at the sequence level [16, 17]. This problem may be overcome by the use of chimeric transgenes assembled from pieces of genomes from the viruses expected to infect the crop [18].

Considerable efforts have been invested to develop virus resistance to many different viruses affecting crops relevant to agriculture and food production in developing countries. The main target crops, besides those mentioned above, have been potato (*Solanum tuberosum* L.) [18, 19, 20, 21], tomato (*Solanum lycopersicum* L.) [22], peppers (*Capsicum annuum* L.) [23, 24], peanuts (*Arachis hypogaea* L.)[25, 26], sugarcane (*Saccharum officinarum* L.) [27], rice (*Oryza* spp.) [28, 29, 30, 31], papaya (*Carica papaya* L.) [32, 33•], passionfruit (*Passiflora edulis* Sims) [34, 35] and soybean (*Glycine max* (L.) Merr.) [36, 37]. However, all have remained at the proof-of-concept stage, and none has passed the regulatory system to the stage of approval for cultivation in developing countries. In fact, only seven transgenic crop species engineered for virus resistance have been approved for cultivation anywhere in the world (Table 1), and only a handful of those have been commercialized. The reasons that these and other virus-resistant transgenic crops have not made it to release in developing countries include a lack of regulatory framework, limited commercial interest and investments, and strong opposition from lobby groups.

**Table 1:** Released transgenic virus-resistant crops approved for cultivation

Event name	Trade name	Gene(s) introduced^a^	Year and country of approval for cultivation
**Bean** **—** ***Phaseolus vulgaris*****L.: 1 Event**			
EMBRAPA 5.1	Not available	BGMV *ac1* (sense and antisense)	2011, Brazil

**Papaya** **—** ***Carica papaya*****L.: 4 Events**			
55-1	Rainbow, SunUp	PRSV_cp	1997, USA 2011, Japan
63-1	Not available	PRSV_cp	1996, USA
Huanong No. 1	Huanong No. 1	PRSV_rep	2006, China
X17-2	Not available	PRSV_cp	2009, USA

**Plum** **—** ***Prunus domestica*****L.: 1 Event**			
C-5	Not available	PPV_cp	2007

**Potato** **—** ***Solanum tuberosum*****L**.**: 15 Events**			
HLMT15-15 HLMT15-3 HLMT15-46	Hi-Lite NewLeaf™ Y potato	PVY_cp	1998, USA
RBMT15-101	New Leaf™ Y Russet Burbank potato	PVY_cp	1998, USA 1999, Canada
RBMT21-129 RBMT21-152 RBMT21-350 RBMT22-082 RBMT22-186 RBMT22-238 RBMT22-262	New Leaf™ Plus Russet Burbank potato	PLRV_orf1 PLRV_orf2	1998, USA 1999, Canada
SEMT15-02 SEMT15-07 SEMT15-15	Shepody NewLeaf™ Y potato	PVY_cp	

**Squash** **—** ***Cucurbita pepo*****L.: 2 Events**			
CZW3	Not available	CMV_cp WMV_cp ZYMV_cp	1996, USA
ZW20	Not available	WMV_cp ZYMV_cp	1994, USA

**Sweet pepper** **—** ***Capsicum annuum*****L.: 1 Event**			
PK-SP01	Not available	CMV_cp	1998, China

**Tomato** **—** ***Solanum lycopersicum*****L.: 1 Event**			
PK-TM8805R (8805R)	Not available	CMV_cp	1999, China

a Viral genes or protein-coding sequences used as transgenes: *ac1*, replicase-encoding gene of bean golden mosaic virus; rep, replicase-encoding sequence of papaya ring sport virus; orf1, replicase-encoding sequence of potato leaf-roll virus (PLRV); orf2, helicase domain-encoding sequence of PLRV; CP, coat protein-encoding sequences of cucumber mosaic virus (CMV), papaya ring spot virus (PRSV), plum pox virus (PPV), potato virus Y (PVY), zucchini yellow mosaic virus (ZYMV) or watermelon mosaic virus (WMV). *Source*: International Service for the Acquisition of Agri-Biotech Applications (ISAAA).

## Approved transgenic crops and their relevance to developing countries

### Transgenic resistance to potato viruses

Potato is the third-most-cultivated food crop in the world after rice and wheat. The climate in highland areas is well suited for potato and cultivation has increased quickly in Africa. Furthermore, root crops such as potato and sweetpotato are expected to suffer less from climate change than many other subsistence crops [38]. Potato virus Y (PVY, a member of the genus *Potyvirus*; family *Potyviridae*), potato leafroll virus (PLRV, a member of the genus *Polerovirus*; family *Luteoviridae*) and potato virus X (PVX, a member of the genus *Potexvirus*; family *Alphaflexiviridae*) are the most common and devastating potato viruses worldwide. The aphid-transmitted PVY and PLRV can cause significant yield losses on their own. The contact-transmitted PVX becomes significant with PVY co-infection, which induces synergistic interactions of the viruses leading to high accumulation of PVX. To solve this problem, virus-derived resistance was engineered to both PVY and PVX in potato cv. Russet Burbank, representing only the second example (after tobacco) of genetically engineered virus resistance in crop plants [39••, 40]. ‘Russet Burbank’ was also engineered for resistance to PLRV, PVY and Colorado potato beetle (*Leptinotarsa decemlineata* Say) in the late 1990s and approved for marketing under the name NewLeaf™ [41, 42].

Potatoes are clonally propagated and thus prone to build up viral infections over generations. In industrial countries, potato viruses are controlled by planting certified virus-free seed potatoes produced under special cultivation schemes. Combined with control of the aphid vectors using pesticides, this has led to a reduction in the prevalence of PLRV over the past 30 years. In low-income countries, however, potato viruses are common and losses are severe, because healthy seed potatoes and pesticides are not commonly available or affordable [43]. Introgression of virus resistance genes to new potato cultivars is demanding and time-consuming due to the highly heterozygous, outcrossing and polyploid nature of potatoes. This combination of factors makes transgenic approaches to virus resistance especially appropriate for potato, as well as other major clonal crops such as bananas, cassava and yam (*Dioscorea* spp.).

Resistance to PLRV in ‘NewLeaf’ reached commercial production in the USA, but the engineered variety remained on the market only a couple of years before it was withdrawn due to the decision by major potato processing industries to abstain from using transgenic potatoes [44]. The demand for ‘NewLeaf’ was not high in the US market, because clean seed can be bought every year and pesticides are affordable, providing alternative means to control viruses. In low-income countries, however, virus-resistant potato varieties would be of major importance in preventing yield losses, because farmers rarely renew their seed potatoes. Resistance against both primary and secondary infection with PLRV has been achieved using efficient inverted repeat hairpin constructs [20]. High levels of resistance in transgenic plants expressing such hairpin constructs have also been obtained against PVY, PVX and the aphid-transmitted potato virus A [18, 21, 45, 19].

The natural dominant PVX resistance gene *Rx*, which confers extreme resistance to a wide spectrum of PVX strains, has been isolated from potato and introduced to PVX-susceptible plants by genetic engineering [46]. Furthermore, virus specificity of the *Rx* gene has been extended by mutation [47^•^]. Thus, there are plenty of opportunities to improve virus resistance in existing potato varieties. Perhaps the ongoing efforts to obtain approval for transgenic late-blight-resistant potato varieties in Uganda and Bangladesh will pave the way for [transgenic] virus resistance in the future.

### Resistance to papaya ringspot virus (PRSV) in papaya

Papaya is a crop predominantly produced and widely consumed in the developing world. It is an important and rich source of essential nutrients, such as vitamins A and C. PRSV (a member of the genus *Potyvirus*; family *Potyviridae*) is a major pathogen of papaya worldwide. PRSV-resistant papaya was one of the first virus-resistant transgenic plants approved (in 1998), and was for a long time the only transgenic crop developed entirely with public funding. PRSV-resistant papaya effectively saved papaya production Hawaii [32, 33•]. However, PRSV resistance in the transgenic papaya is rather specific to local strains, leaving Hawaii vulnerable to the introduction of new PRSV strains. PRSV-resistant papayas have since been developed using improved transgene construct design and tested against local strains in other countries, including Brazil, China, Jamaica, Venezuela, Taiwan, Thailand and The Philippines. However, the transgenic cultivars have not been approved for cultivation in any of these countries. Davidson [48] published an illustrative case study concerning attempts to introduce PRSV-resistant papaya in Thailand, highlighting the complexity and controversy surrounding adoption of genetically modified crops in lower-income countries.

### Viruses in common bean

Common beans (*Phaseolus vulgaris* L.) and vegetable crops such as tomato, peppers and cucurbits (*Cucurbita* spp.) are damaged by whitefly-transmitted begomoviruses worldwide [49, 50] and particularly in Latin America [51]. Bean golden mosaic virus (BGMV) and the related bean yellow golden mosaic virus are among the biggest constraints on bean production in Latin America. After almost two decades of work, the Brazilian Agricultural Research Corporation (EMBRAPA) was able to produce a transgenic line of common bean showing high and stable levels of resistance to BGMV [52]. The transgenic line EMBRAPA 5.1 was approved for cultivation in 2011, and field trials for registration of several new cultivars developed from EMBRAPA 5.1 by breeding were initiated in 2012 [53]. The resistance generated is expected to enable recovery of bean production in BGMV-affected areas, increase yields and quality, and to reduce the need for pesticide applications for vector control in Brazil. These transgenic lines may not necessarily confer resistance to bean-infecting begomoviruses in other parts of the world due to differences among viruses and virus strains. Nonetheless, the approach using an inverted repeat construct targeting the viral replicase gene and a highly efficient transformation system can be applied to generate bean varieties that are resistant to the main begomoviruses found in other developing countries.

## Transgenic virus resistance specifically aimed at low-income countries

Although no plants genetically engineered for virus resistance have been approved for cultivation in low-income countries, many efforts toward this end are ongoing, particularly in Africa where recurring virus epidemics are a major constraint on crop production. These efforts are focused primarily on major staple and food security crops, such as cassava, sweetpotato, banana, rice and maize.

### Cassava mosaic and cassava brown streak diseases

Cassava (*Manihot esculenta* Crantz) is very important as a subsistence crop in Africa. The cassava mosaic epidemic started and spread quickly in East Africa in the mid-1990s, devastating cassava crops entirely in many regions. The disease escalated when African cassava mosaic virus and virulent recombinants with other whitefly-transmitted cassava-infecting begomoviruses co-infected cassava plants, resulting in synergism, very severe symptoms, growth retardation and new virulent recombinants of the viruses [2]. More mosaic-disease-resistant cassava germplasm identified in West Africa was introduced to breeding programs, and the new varieties eventually slowed down the epidemic [54]. However, the risk of new epidemics remains, and new means offered by biotechnology are being used in resistance breeding [54, 55, 56].

Cassava brown streak disease (CBSD) is caused by two closely related whitefly-transmitted ipomoviruses [6]. Both viruses cause similar symptoms, namely severe necrosis in the storage roots making them inedible, but only mild symptoms in leaves. CBSD was reported in the coastal area of East Africa in the 1930s, but a new major epidemic started in Tanzania, Uganda and Kenya in the mid-1990s [5]. Local resistance breeding programs have increased tolerance to symptom formation in new cassava varieties, but resistance to the brown streak viruses has not yet been achieved. However, transformation of the Ugandan farmer-preferred cassava cultivar TME with a virus-derived inverted repeat construct appears effective against both brown streak viruses [57], but inadvertently resulted in the loss of resistance of CMD by the *CMD2* gene, apparently as an unexpected consequence of the somatic embryogenesis process involved in regenerating transgenic plants [58].

### Sweetpotato virus disease

Sweetpotato (*Ipomoea batatas* Lam.) originates from South and Central America, but has a particularly significant role as a subsistence crop in Africa [43]. The importance of the sweetpotato increased during the aforementioned CMD epidemic in 1990s. Sweetpotato is a generally healthy crop, suffering from only a few diseases. Over 30 viruses infect sweetpotatoes, but most of them cause mild or no symptoms and only minor yield losses. The main disease is SPVD, which is characterized by severe symptoms of leaf malformation and stunted plant growth. Diseased plants may fail to produce any tuberous roots for consumption. SPVD develops in sweetpotato plants co-infected with the whitefly-transmitted SPCSV and virtually any other sweetpotato virus [8, 59, 60, 61]. Targeting SPCSV with pathogen-derived resistance using various genomic regions of SPCSV as transgenes significantly reduces accumulation of SPCSV in transgenic sweetpotato plants, but infection of these plants with other sweetpotato viruses breaks down resistance and induces severe symptoms [62]. Studies indicate that the double-stranded-RNA-specific RNase III enzyme of SPCSV suppresses antiviral RNAi by cleaving the small interfering RNAs employed to target viral RNA degradation [60]. Attempts to generate resistant plants by expression of dominant negative mutants of SPCSV RNase III have been unsuccessful so far, but efforts continue (J. Kreuze and J. Valkonen, unpublished).

### Banana bunchy top disease

Banana bunchy top virus (BBTV) is transmitted by aphids and is the most destructive viral pathogen of bananas and plantains worldwide. It originates in Asia, was introduced to Africa probably from the South Pacific [63, 64], and has now reached most areas of sub-Saharan Africa [61]. BBTV is currently a major food security concern, because infected bunches are difficult to recognize by smallholder farmers and will yield no usable fruit. Shekhawat *et al.* [65] were able to generate high levels of resistance to BBTV by targeting the viral replicase gene with intron-hairpin-RNA transcripts. This approach was also efficient against other virus members of the family *Nanoviridae*.

The challenge in introducing transgenic resistance to different banana cultivars lies in the sterility of the vegetatively propagated cultivars and the need to transform each cultivar separately, which is not a trivial task. On the other hand, as banana cultivars are essentially sterile, transgene flow to other cultivars or wild *Musa* species is unlikely. Since copy numbers and integration sites of transgenes are difficult to control, application of modern genome-editing technologies could allow targeting of specific host genes [66], for example, those playing a role in virus susceptibility, and achieving virus resistance.

### Viruses of rice and maize

Rice yellow mottle virus (RYMV, a member of the genus *Sobemovirus*) causes a major disease in rice that is used as an example of the key role of agricultural intensification in plant virus emergence [67]. The recessive resistance genes in rice germplasm can be overcome by the virus [68]. As early as 1998, however, highly efficient resistance generated by expression of open reading frame 2 of RYMV was achieved in transgenic lines of African rice varieties. Resistance remained stable over at least three generations and conferred resistance to a wide range of RYMV isolates [31]. Because rice is seed-propagated, the transgenic resistance trait can be introgressed into local varieties via crossing. However, to our knowledge, these lines never progressed beyond the proof-of-concept stage.

MSV causes maize streak disease, a major constraint of maize production in Africa [69]. Dominant and recessive natural resistance genes are available for protecting crops against MSV. Furthermore, engineered resistance to MSV is available in transgenic maize plants by expression of a defective form of a viral gene involved in viral replication [70] and was the first transgenic crop plant developed in Africa. A better transgene expression system activated only by MSV infection is under preparation [71].

Maize (corn) lethal necrosis caused by co-infection of maize chlorotic mottle virus and another potyvirus, maize dwarf mosaic virus was described in USA, but is currently ravaging East-Africa and threatening to spread further across the continent. The disease can be controlled by transgenic resistance against maize dwarf mosaic virus [72], which could provide a solution.

## Naturally transgenic virus resistance?

Recently, natural transgenes corresponding to the transfer DNAs (T-DNA) of *Agrobacterium* spp. were found integrated in the sweetpotato genome, which raises a question about their possible role in host defense or crop domestication [73^••^]. T-DNAs of *Agrobacterium* are also found in other plant species [74], analogous to transgenic plants created by *Agrobacterium*-mediated transformation. It is more common, however, to find viral sequences integrated in plant genomes, which is comparable to plants transformed by particle bombardment and results in random integration of DNA in the plant genome.

**Figure 1:**
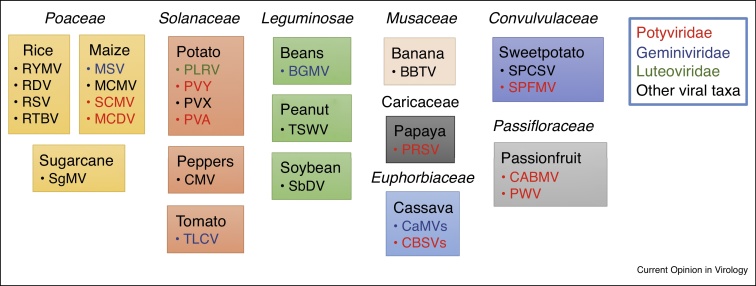
Tropical crops engineered for virus resistance. The boxes are colored according to the plant family. Each box contains one crop species and the acronyms in the box indicate the virus(es) against which resistance was engineered. Viruses with acronyms of the same color are members of the same family. The viruses highlighted in bold are discussed in this paper. BGMV, bean golden mosaic virus; BSV, banana streak virus; CABMV, cowpea aphid borne mosaic virus; CBSVs, cassava brown streak virus and Ugandan cassava brown streak virus; CMV, cucumber mosaic virus; CaMVs, cassava mosaic viruses; MSV, maize streak virus; MCMV, maize chlorotic mottle virus; PWV, passionfruit woodiness virus; RDV, rice dwarf virus; RSV, rice stripe virus; RTBV, rice tungro bacilliform virus; RYMV, rice yellow mottle virus; SbDV, soybean dwarf virus; SCMV, sugar cane mosaic virus; SgMV, sorghum mosaic virus; SPCSV, sweetpotato chlorotic stunt virus; SPFMV, sweet potato feathery mottle virus; TLCV, tomato leaf curl virus; TSWV, tomato spotted wilt virus.

Integration of viral sequences in a plant genome was first realized in banana carrying fragments of banana streak virus, a pararetrovirus with a double-stranded DNA genome encapsidated in bacilliform particles [75^••^], and later with other pararetroviruses. For example, petunia vein clearing virus — like and tobacco vein clearing viru*s* — like sequences are found integrated in petunia and many solanaceous crops, respectively. Viral integrations have been observed also in woody plants such as grapevine and fig [76, 77, 78, 79]. Some of the integrated sequences may be reactivated and cause disease when plants are affected by stress or other exceptional circumstances, such as tissue culture or interspecific crosses. However, pararetroviruses are mostly dormant and the host plants are rather resistant to them; indeed, retroviruses integrated in the plant genome may confer resistance against infecting homologous viruses [78]. The most likely mechanism of resistance is RNA silencing induced against the endogenous sequences as a method to control viral expression. Sequences of other DNA and RNA viruses are also found integrated in plant genomes [80^•^]. The integrated sequence of cucumber mosaic virus in soybean is structurally similar to the hairpin-RNA constructs designed to induce target-specific RNA silencing and virus resistance [81] (Figure 1).

## Concluding remarks

Taken together, the recently adopted methods for genome editing, such as CRISPR-Cas9, are directly adopted from living organisms and, hence, incompatible with the traditional definitions of genetic engineering and transgenic plants. It is also apparent that RNA silencing against virus-derived sequences occurs naturally in plants on a large scale based on viral sequences integrated into plant genomes. It could be time to reconsider what is ‘transgenic’ and requires regulation. Reassessment of the rules and regulations could conclude that deregulation of virus resistance mediated by homology-based RNA silencing might be reasonable.

## References and recommended reading

Papers of particular interest, published within the period of review, have been highlighted as:

• of special interest
•• of outstanding interest
